# Common neural correlates of real and imagined movements contributing to the performance of brain–machine interfaces

**DOI:** 10.1038/srep24663

**Published:** 2016-04-19

**Authors:** Hisato Sugata, Masayuki Hirata, Takufumi Yanagisawa, Kojiro Matsushita, Shiro Yorifuji, Toshiki Yoshimine

**Affiliations:** 1 Department of Neurosurgery, Osaka University Medical School, 2-2 Yamadaoka, Osaka, 565-0871, Japan; 2 Faculty of Welfare and Health Science, Oita University, 700 Dannoharu, Oita, 870-1192, Japan; 3 Division of Functional Diagnostic Science, Osaka University Graduate School of Medicine, 1-7 Yamadaoka, Osaka, 565-0871, Japan; 4 ATR Computational Neuroscience Laboratories, 2-2-2 Hikaridai, Keihannna Science City, Kyoto, 619-0288, Japan; 5 Department of Mechanical Engineering, Gifu University, 1-1 Yanagido, Gifu, 501-1193, Japan

## Abstract

The relationship between M1 activity representing motor information in real and imagined movements have not been investigated with high spatiotemporal resolution using non-invasive measurements. We examined the similarities and differences in M1 activity during real and imagined movements. Ten subjects performed or imagined three types of right upper limb movements. To infer the movement type, we used 40 virtual channels in the M1 contralateral to the movement side (cM1) using a beamforming approach. For both real and imagined movements, cM1 activities increased around response onset, after which their intensities were significantly different. Similarly, although decoding accuracies surpassed the chance level in both real and imagined movements, these were significantly different after the onset. Single virtual channel-based analysis showed that decoding accuracy significantly increased around the hand and arm areas during real and imagined movements and that these are spatially correlated. The temporal correlation of decoding accuracy significantly increased around the hand and arm areas, except for the period immediately after response onset. Our results suggest that cM1 is involved in similar neural activities related to the representation of motor information during real and imagined movements, except for presence or absence of sensory–motor integration induced by sensory feedback.

Brain–machine interfaces (BMIs) translate brain signals, which are recorded using invasive or non-invasive measurement techniques, into commands that can control external devices such as prosthetic arms and computers^1^,^2^,^3^. This technology is expected to offer patients who have lost control of voluntary movements, including those with amyotrophic lateral sclerosis (ALS) and spinal cord injury, greater independence and an improved quality of life. Such improvements can be achieved with the use of external devices to communicate with others and manipulate the environment^4^,^5^.

Signals recorded from the primary motor cortex (M1) play a significant role in controlling external devices^6^,^7^,^8^. Recently, the importance of M1 signals for BMIs has been demonstrated for real and imagined movements using various types of signal platforms, such as electroencephalography (EEG)^9^,^10^, magnetoencephalography (MEG)^11^,^12^,^13^,^14^,^15^,^16^,^17^, and electrocorticography (ECoG)^18^,^19^,^20^,^21^. However, for imagined movements, the involvement of M1 activation remains controversial. A previous study reported that M1 is not necessary to perform an imagined movement^22^. Several other studies have not detected M1 activation or have shown only transient activation during imagined movements^23^,^24^. However, using direct cellular recordings, Georgopoulos *et al.*^25^ have demonstrated that M1 activity contributes to motor imagery. Some recent studies that used invasive^26^ or non-invasive methods^27^,^28^,^29^,^30^ also reported that imagined movements activate M1. In addition, we recently reported that the strength of functional connectivities between M1 and the motor association area affects the performance of BMIs in both real and imagined movements^31^. Furthermore, M1 activity during imagined movements has been recorded not only in healthy subjects but also in patients with stroke^21^, tetraplegia^6^,^7^,^32^, and ALS^33^. To the best of our knowledge, no studies have focused on the relationship between M1 activity representing motor information in real and imagined movements with high spatiotemporal resolution using non-invasive measurements. Considering the above findings, we hypothesized that there are common neural responses in M1 that are significantly active during both real and imagined movements, and may therefore represent promising target signals for use with BMIs.

The aim of this study was to examine the similarities and differences in M1 activity during real and imagined movements in terms of neural decoding. Thus, we used MEG, which has several advantages in the analysis of neurophysiological signals compared with EEG and functional magnetic resonance imaging (fMRI). MEG has higher spatial resolution than EEG, and it can record a direct correlate of neural activity with a higher temporal resolution than fMRI. Spatially filtered M1 signals were extracted using a beamforming approach to classify the three types of unilateral upper limb movements in real and imagined movements on a single-trial basis, and the correlation of decoding accuracy between the two movements was examined.

## Results

### Intensities of contralateral M1 activities and decoding accuracy between real and imagined movements

**Figure 1:**
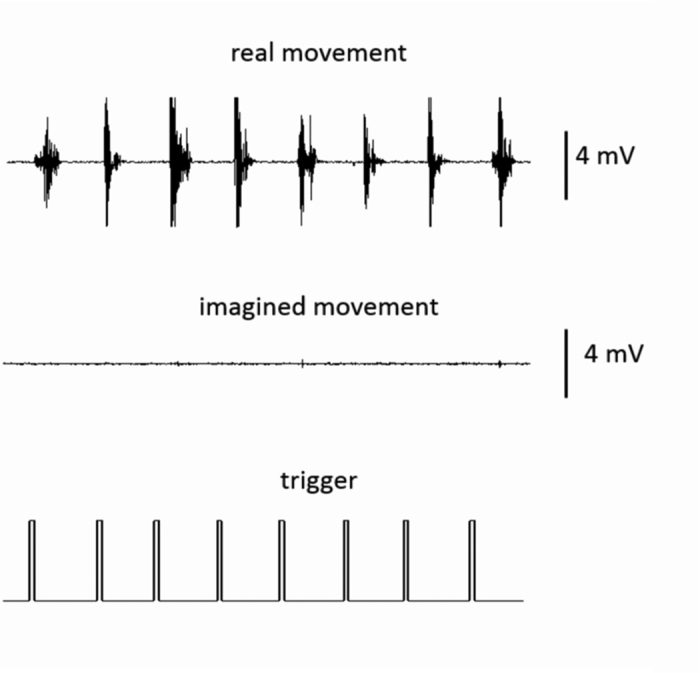
A representative electromyogram amplitude during real and imagined movements. Robust muscle activities were observed during the real movement, but not during imagined movement, after the presentation of the execution cue (trigger).

Electromyogram (EMG) showed a difference in response between real and imagined movements (Fig. 1). Muscle activities were observed during the real movement but not during the imagined movement. There was no relationship between the EMG signals recorded during real and imagined movements (Fig. S1).

**Figure 2:**
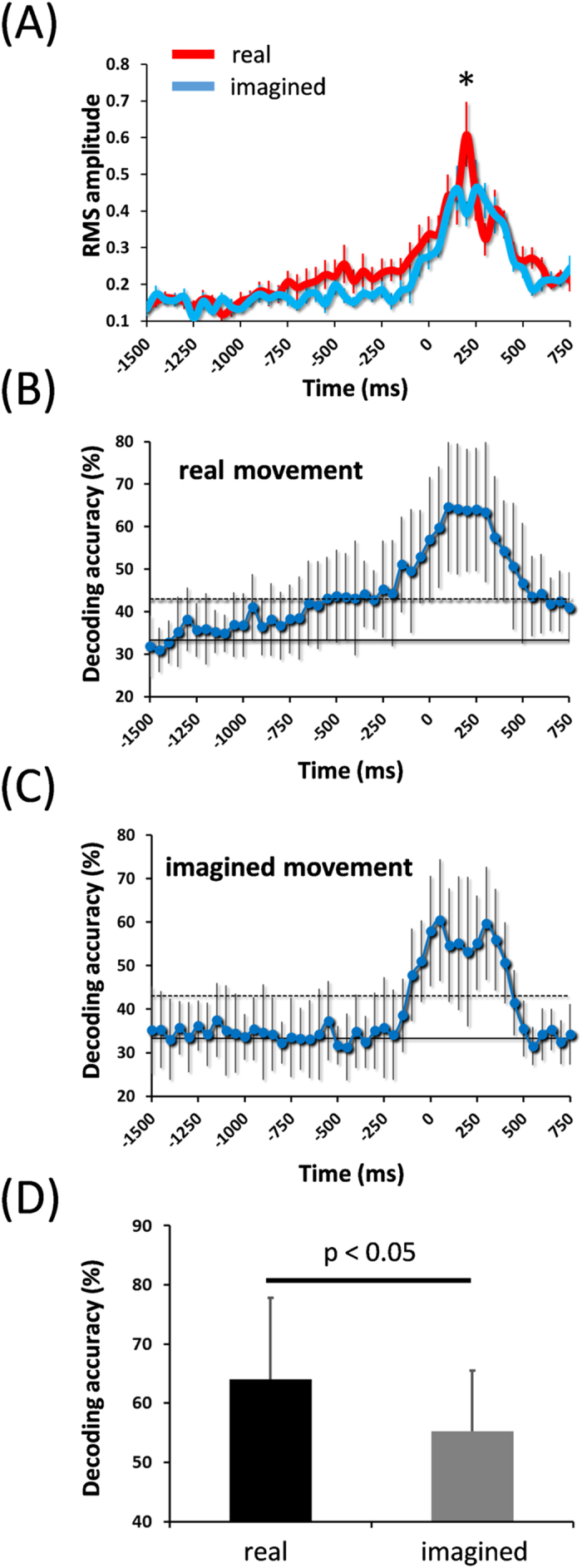
Intensity of cM1 activity and decoding accuracy during real and imagined movements. (**A)** cM1 activity sharply peaked at 200 ms in the real movement, whereas only small peaks were observed at 150 and 250 ms in the imagined movement. A significant difference in the intensity of cM1 activity between real and imagined movements was observed at 200 ms (Mann–Whitney U-test, *p < 0.01) (error bar, standard error). (**B**) In the real movement, decoding accuracy gradually increased before response onset and peaked at 100 ms (error bar, SD). Decoding accuracy was plotted for the first sample acquired in the time window. The two horizontal solid and dotted lines indicate decoding accuracy at the chance level (33.3%; binomial test, *p *= 0.01 for both). (**C)** In the imagined movement, decoding accuracy showed two peaks at 50 and 300 ms (binomial test, *p* < 0.01). (**D)** Significant differences in decoding accuracy between real and imagined movements were observed at 200 ms (Mann–Whitney U-test, *p* < 0.05).

Figure 2A shows the time courses of brain activities in M1 contralateral to the movement side (cM1) during real and imagined movements. For real movements, the intensity of cM1 activity gradually increased from −500 ms and sharply peaked at 200 ms, whereas no such clear peak was observed for the imagined movement. Instead, two small peaks were observed at 150 and 250 ms. A significant difference in the intensity of cM1 activity between real and imagined movements was observed at 200 ms (Mann–Whitney U-test, *p* < 0.01) (Fig. 2A). To compare cM1 activity with other brain regions, the intensities of brain activity were also calculated from the following seven regions of interest (ROIs); contralateral S1 (cS1), frontal (c-frontal), and parietal (c-parietal) areas; ipsilateral M1 (iM1), S1 (iS1), frontal (i-frontal), and parietal (i-parietal) areas. However, there were no significant differences between the real and imagined movements in these brain regions (Fig. S2).

Using the normalized amplitudes of neuromagnetic activity, decoding accuracy was calculated at each time point in all cM1 virtual channels. For real movements, decoding accuracy gradually increased from −500 ms and peaked at 100 ms (binomial test, *p* < 0.01) (Fig. 2B). The peak decoding accuracy averaged over subjects reached 64.6 ± 15.2% (mean ± SD). For imagined movements, decoding accuracy increased at −100 ms, and two peaks were obtained at 50 and 300 ms (binomial test, *p* < 0.01) (Fig. 2C). The peak decoding accuracy averaged over subjects was 60.4 ± 13.9% at 50 ms. A significant difference in decoding accuracy between real and imagined movements was observed at 200 ms (*p* < 0.05, Mann–Whitney U-test) (Fig. 2D). To examine the representation of motor information in other brain regions, decoding accuracies were calculated from the other seven ROIs. The results showed that the second largest increase in decoding accuracy after that in cM1 was in cS1 (Fig. S3). Decoding accuracies were also calculated for real and imagined movements in the following frequency bands: alpha (8–13 Hz), beta (13–25 Hz), low gamma (25–50 Hz), and high gamma (50–100 Hz). Clear event-related desynchronizations (ERDs) were observed in the alpha, beta, and low gamma bands before response onset for both real and imagined movements, and event-related synchronization (ERS) was observed in the high gamma band which corresponded to onset for real movement (Fig. S4). However, none of these decoding accuracies were significant in cM1 (Fig. S5), or the other seven ROIs (Fig. S6).

**Figure 3:**
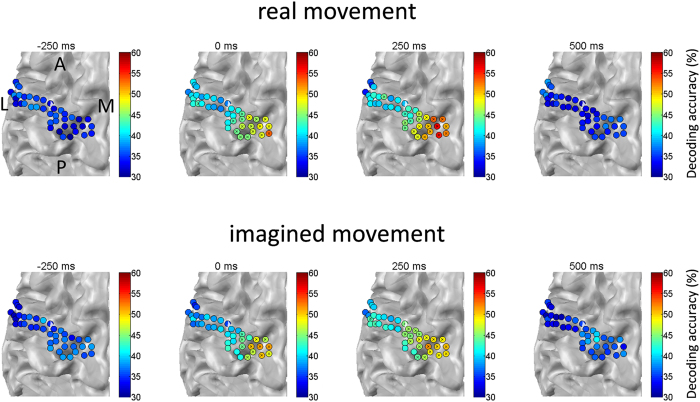
Spatial distribution of decoding accuracies averaged over subjects. Significant decoding accuracies were observed after response onset at the medial part of cM1, particularly around hand and arm areas, during both real and imagined movements. Decoding accuracy was plotted for the first sample acquired in the time window. Virtual channels with significant accuracies are marked with an “x” (binomial test, *p* < 0.01). A, anterior; L, lateral; M, medial; P, posterior.

In addition, the decoding analysis based on a single virtual channel showed a gradual and significant increase of decoding accuracy around the medial part of cM1 in line with response onset in both real and imagined movements (binomial test, *p* < 0.01) (Fig. 3 and Supplementary movie 1, 2). Further, decoding accuracy increased around the medial part of cS1 corresponding to the response onset for both real and imagined movements (Supplementary movie 3 and 4).

In order to rule out the possibility that EMG activity may affect decoding accuracy during real and imagined movements, we examined their relationship in cM1. The results showed that there were no significant correlations between decoding accuracy and EMG activity for either movement (Fig. S7).

### Correlations of decoding accuracy between real and imagined movements

**Figure 4:**
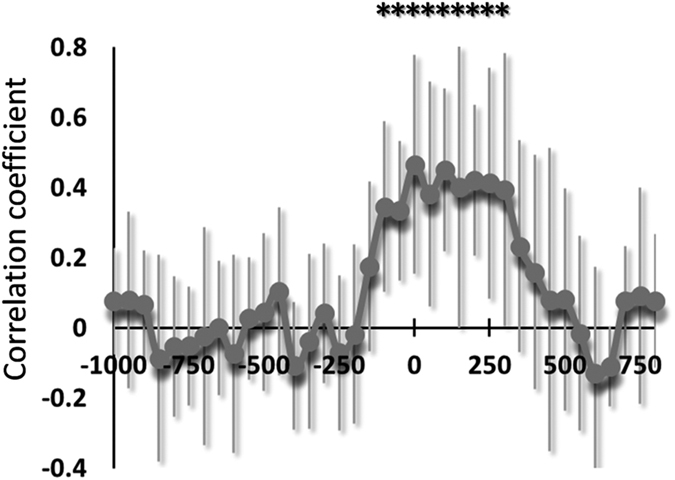
Spatial correlation of decoding accuracies between real and imagined movements averaged over subjects. Significant spatial correlations were observed around response onset (Pearson’s test, *p < 0.05) (error bar, SD).

To examine the relationship between cM1 activity representing motor information in real and imagined movements, we calculated the spatial correlation of decoding accuracy between both movements at each time point. Figure 4 depicts the time course of the averaged spatial correlations. The correlation coefficients significantly increased from −100 to 300 ms (Pearson’s correlation test, *p* < 0.05). A similar result was also observed in cS1 from 100 to 300 ms but not in the other six ROIs (Fig. S8).

**Figure 5:**
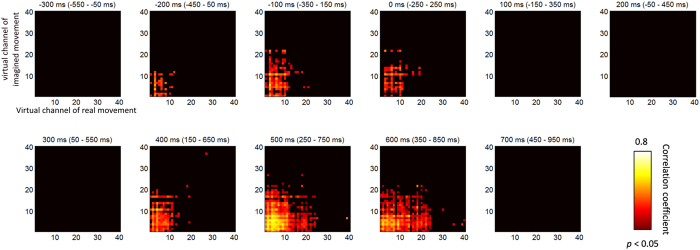
Temporal correlation of decoding accuracy between real and imagined movements. Each plot depicts the correlation coefficients averaged over subjects at each time window. Temporal correlation began to increase significantly from −200 ms (from −450 to 50 ms) around the medial part of cM1, particularly around the hand and arm areas. These significant correlations disappeared around response onset and reappeared from 400 ms (150–650 ms). Correlation coefficients at *p* < 0.05 were considered statistically significant and were plotted (Spearman’s rank correlation test, *p* < 0.05, false discovery rate-corrected).

**Figure 6:**
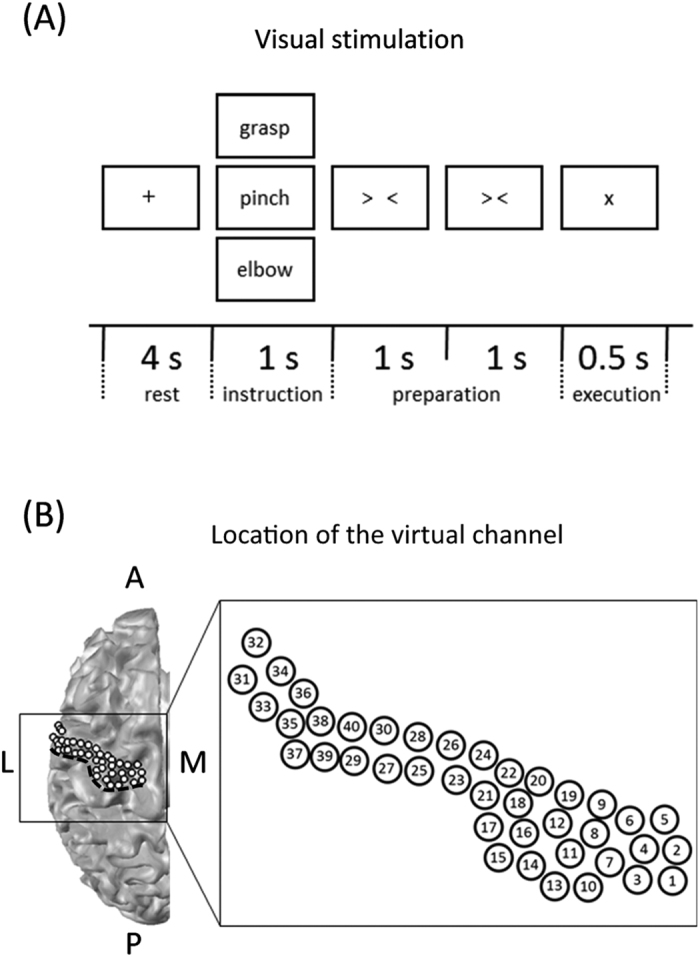
Task paradigm and location of virtual channels. (**A)** Experimental paradigm. Subjects performed the motion and imagined tasks in the same sequence. The trial consisted of four phases; the rest phase, instruction phase, preparation phase, and execution phase. In the rest phase, a black fixation cross “+” was presented for 4 s. Subjects fixed their eyes on the cross. In the instruction phase, a Japanese word representing one of three movements was presented for 1 s. Then, in the preparation phase, two timing cues, “> <” and “> <,” were presented one at a time, each for 1 s to aid the subjects in preparing for the execution of real or imagined movements. In the execution phase, subjects performed the real or imagined movement, as requested in the instruction phase stage, after the appearance of the execution cue “×.” Each of the three movements was performed 60 times. (**B)** Locations of the virtual channels are indicated by white dots on a three-dimensional brain model. Forty virtual channels were located on the left cM1 at intervals of 2.5 mm. The black dotted line indicates the location of the central sulcus. A, anterior; L, lateral; M, medial; P, posterior.

The temporal correlation of decoding accuracy between real and imagined movements was also examined in each virtual channel. Figure 5 shows temporal correlations of particular time ranges of decoding accuracy in cM1 between real and imagined movements. The temporal correlation significantly increased from −200 ms (−450–50 ms) around the medial part of cM1, including the hand and arm areas [Spearman’s rank correlation test, *p* < 0.05, false discovery rate (FDR)-corrected] (Fig. 5, also see Fig. 6B). Although this significant correlation disappeared around response onset, it reappeared from 400 ms (150–650 ms) (Fig. 5 and Supplementary movie 5). Temporal correlations were also calculated in the other seven ROIs. The results showed a significant correlation between cS1 before and after response onset but not from 100 to 300 ms, which was weaker than that in cM1 (Fig. S9) (Spearman’s rank correlation test, *p* < 0.05, FDR-corrected). These significant correlations tended to cluster around the hand and arm areas. The remaining six ROIs showed no significant temporal correlation.

## Discussion

Many previous studies have used invasive or non-invasive measurements to demonstrate the importance of cM1 activity for decoding movement types, directions, and trajectories during real and imagined movements^6^,^7^,^20^,^34^. Recently, we demonstrated the effect of functional connectivity between cM1 and motor association areas on BMI performance during real and imagined movements using MEG^31^. To the best of our knowledge, no previous studies have investigated the relationship between cM1 activity representing motor information in real and imagined movements with high spatiotemporal resolution using non-invasive measurements. In this study, we performed neurophysiological and computational analyses to reveal the common neural correlates of cM1 activity related to motor representation using MEG.

The decoding accuracy significantly increased in line with the increase in cM1 activity for both real and imagined movements. Because there was no significant correlation between decoding accuracy and EMG activity, movement types were thought to be decoded only by cM1 signals. This result is consistent with a previous study, which reported that cM1 activity reflecting corticospinal motor output from the cM1 to the periphery was related to BMI performance^6^,^7^,^8^,^20^,^21^,^35^,^36^. In addition, our single virtual channel-based decoding analysis showed significant decoding accuracy around the hand and arm areas for both real and imagined movements after response onset, and these spatial distributions significantly correlated. Furthermore, the decoding accuracy between real and imagined movements showed a significant temporal correlation from −200 to 0 ms and 400 to 600 ms. The significant correlation from −200 to 0 ms shows a similar fluctuation in cM1 activity representing the movement type in both real and imagined movements. The correlation from 400 to 600 ms may reflect a similar decrease in cM1 activity at the end of both real and imagined movements (i.e., a similar decrease in decoding accuracy). Notably, decoding accuracy significantly surpassed the chance level for both real and imagined movements, and these spatial and temporal correlations were mainly observed around the hand and arm areas. These results suggest that the neural substrates involved in representation of movement type over cM1 overlap spatially and temporally. In other words, the cM1 somatotopic information needed to decode imagined movement may be just as useful as that required to decode real movement. Thus, our results support previous studies which showed that cM1 activity was useful for decoding both movement types^6^,^8^,^10^,^14^,^21^.

The second largest increase in brain activity and decoding accuracy after that in cM1 was observed in cS1 for both real and imagined movements. Our single virtual channel-based decoding analysis showed a significant increase in the decoding accuracy around the hand and arm areas for both movements. In addition, spatiotemporal correlations were also found in cS1 as well as cM1. Previous studies have suggested that motor commands descending from M1 to the spinal cord are collaterally forwarded to the sensory system^37^,^38^. This collateral descending output is termed “efference copy”. When the efference copy reaches sensory areas, the evoked activity similar to that of sensory feedback expected from the movement, has been reported^39^,^40^,^41^,^42^. Other studies have demonstrated overlapping sensory and motor representations in rodents^43^ and corticospinal neurons in the monkey S1^44^,^45^. Furthermore, a recent study has provided evidence for a direct role of the sensory cortex in motor control using rodents^46^. These findings suggest the M1 and S1 are strongly coupled when integrating sensory-motor information. Considering that our result showed robust brain activity and high decoding accuracy in cS1 for both real and imagined movements, important information related to motor representation may be included in both cM1 and cS1.

Contrary to the common aspects mentioned above, cM1 activity and decoding accuracy temporarily differed between real and imagined movements after response onset. Corresponding to this period, the temporal correlation of decoding accuracy between real and imagined movements also disappeared. This period is consistent with the latency of sensory feedback from the periphery to cS1 during voluntary movement^47^. Previous studies have reported that the anterior part of M1 is associated with movement, while the posterior part is activated by sensory inputs^48^,^49^ because of its abundant somatosensory afferents^50^. In addition, some non-primate studies have shown that M1 and S1 are reciprocally connected and that the synaptic inputs from S1 to M1 are stronger than those from M1 to S1^51^. Furthermore, sensory-evoked activity is first presented to S1 and then propagated to M1^52^, indicating that the sensory-motor connection plays an important role in integrating sensory-motor information^53^,^54^. Considering that M1 reciprocally connects with S1 and also receives somatosensory input from it^52^ and muscle spindles^55^, the deficiency of somatosensory feedback resulting from a lack of voluntary movement may affect sensory-motor integration^56^,^57^,^58^. Thus, our results suggest that the significant differences in cM1 activity and decoding accuracy just after response onset are related to the presence or absence of sensory-motor integration that results from somatosensory feedback. Temporal characteristics similar to those in cM1 were also observed in cS1, which means that significant temporal correlations around the hand and arm areas in cS1 temporarily disappear after response onset. This result may also stem from the presence or absence of sensory feedback and subsequent sensory-motor integration with real and imagined movements.

The decoding accuracy also increased in c-frontal and c-parietal areas near cM1 and cS1. These regions were previously reported to be involved in the representation of movement-related information^59^,^60^,^61^. However, our results showed that there were no significant spatiotemporal correlations in decoding accuracy between real and imagined movements, suggesting that the neural mechanisms of movement type representation may differ between real and imagined movements in these brain regions.

In the present study, we also calculated the decoding accuracy using frequency powers. However, no significant decoding accuracy was obtained in cM1 or other seven ROIs. Previous studies using non-invasive measurements have successfully classified a moving state from a resting state using frequency band powers in the sensorimotor cortex^10^,^12^,^62^, whereas these features have been unsuited for inferring multiple movement types^15^. A recent study using MEG also showed that frequency powers were not capable of extracting motor information about movement type^63^. In contrast, previous studies using ECoG demonstrated that frequency band powers, particularly the high gamma band, were informative^19^,^20^, suggesting that our decoding results using frequency band powers may reflect a lower signal-to-noise ratio because of non-invasive MEG measurements for the high gamma band^15^. On the other hand, we obtained significantly high decoding accuracy using the averaged normalized amplitudes of MEG signals. This feature mainly comprises the low frequency components of the signals extracted by averaging within sliding time windows. The low frequency signal component is reportedly suitable for decoding movement trajectories^19^,^64^, types^8^,^14^,^35^,^36^, and directions^11^,^15^. Given that the low frequency signal components have higher signal-to-noise ratios than the high frequency components, the decoding feature used in this study may have been suitable for classifying the unilateral upper limb movements, especially for MEG measurements.

In summary, cM1 neural activity representing movement type was similar for real and imagined movements, meaning that the measurement of cM1 activity is useful for decoding movement type. On the other hand, the intensity of activity and decoding accuracy in cM1 temporarily differed after response onset between real and imagined movements. Given that M1 reciprocally connects with S1 and receives somatosensory input from S1 and muscle spindles, a lack of sensory-motor integration resulting from deficient somatosensory feedback may affect the significance of activity and decoding accuracy differences in cM1 between real and imagined movements just after response onset. Previous studies have reported that BMI training combining proprioceptive sensory feedback changes cM1 activity^65^ and facilitates functional recovery in stroke patients^66^, suggesting that the performance of imagery-based BMIs can be improved by utilizing appropriate somatosensory feedback. The absence of somatosensory feedback in current BMIs limits the quality of the movements^67^. Thus, understanding the interaction between efferent and afferent signals in M1 and S1 during real and imagined movements is essential for the development of high-quality BMIs and their application in various clinical fields, such as neurorehabilitation. Further investigation may lead to the establishment of a preoperative evaluation method using invasive BMIs and their application in clinical settings.

## Materials and Methods

### Ethics statement

This study was conducted in accordance with the protocol approved by the Ethics Committee of Osaka University Hospital (approval number 11125-4). Informed consent was obtained from all subjects prior to participation.

### Subjects

We enrolled 10 healthy volunteers (five males and five females; mean age: 25.8 years, standard deviation (SD): 7.4, age range: 21–35 years). All subjects were confirmed to be right-handed using the Edinburgh Handedness Inventory (EHI)^68^. All subjects had an EHI score of 100, no history of neurological or psychiatric diseases, and normal vision.

### Tasks

The experimental paradigm is shown in Fig. 6A. Participants performed two tasks, a motion task and an imagined task. We previously demonstrated the contribution of cM1 signals in the classification of movement types using the same motor task, on the basis of ECoG^8^ and MEG results^14^. An epoch began with 4 s in the rest phase, and a black fixation cross to fix the eyes of the subject on the screen was presented. Then, a Japanese word representing one of the three right upper limb movements (grasping, pinching, or elbow flexion) was presented to instruct the subject on which movement to perform or imagine after the execution cue appeared. Two timing cues, “> <” and “> <,” were subsequently presented one at a time, each for 1 s, to aid the subjects in preparing for the execution of real or imagined movements. In both the motion and imagined tasks, subjects were instructed to perform the requested movement immediately after the execution cue appeared. Each of the three types of movements was performed 60 times during real movement trials, and the movement in any given epoch was randomly selected. Then the imagined motor tasks were conducted in the same manner. Because we believed that performing real movements first would make it easier to perform the imagined movement, motion tasks were conducted before imagined tasks. To minimize fatigue, there was a minimum 30-min interval between the motion and imagined tasks.

### MEG measurements

Neuromagnetic activity was recorded in a magnetically shielded room using a 160-channel whole-head MEG system equipped with coaxial type gradiometers (MEG vision NEO, Yokogawa Electric Corporation, Kanazawa, Japan). Subjects lay in supine position with their head centered. The position of the head was measured before and after the recording, using five coils that were placed on the face (the external meatus of each ear and three points on the forehead). Using a visual presentation system and a liquid crystal projector, visual stimuli were displayed on a projection screen positioned 325 mm from the subject’s eyes (Presentation, Neurobehavioral Systems, Albany, CA, USA; LVP-HC6800, Mitsubishi Electric, Tokyo, Japan). Data were sampled at a rate of 1000 Hz with an online low-pass filter at 200 Hz. To reduce artifacts from muscle activity and eye movements, we instructed the subjects to rest their elbows on a cushion, to avoid shoulder movements, and to observe the center of the display without ocular movements or excessive blinking. To monitor unwanted muscular artifacts, muscle activity was simultaneously recorded on an electromyogram (EMG), with electrodes positioned on the flexor pollicis brevis, flexor digitorum superficialis, and biceps brachii muscles.

We also acquired structural MRIs, obtained using a 3.0 T MRI system (Signa HDxt Excite 3.0 T, GE Healthcare UK Ltd., Buckinghamshire, UK). To align MEG data with individual MRI data, three-dimensional facial surfaces were superimposed onto the anatomical facial surface provided by individual MRI data, with an anatomical accuracy of a few millimeters.

### Virtual channels and preprocessing

After data acquisition, a 60-Hz notch filter was applied to eliminate AC line noise. Eye-blink artifacts were separated and eliminated using signal-space projection, a method for separating external disturbances, implemented in Brainstorm (http://neuroimage.usc.edu/brainstorm)^69^.

To extract M1 contralateral to the movement side (cM1) signals from the MEG sensor, we used an adaptive, spatial filtering beamforming technique^70^. This approach is used to estimate the temporal course of neural activity at a particular site in the brain marked by an imaging voxel such as that derived from MRI. The output of such a spatial filter is termed a virtual channel or virtual sensor^71^. The beamformer is constructed to project signals exclusively from targeted voxels while eliminating residual noise to suppress signals from other parts of the brain. Thus, virtual channels provide data regarding neural activity at target voxels with a considerably higher signal-to-noise ratio than that of raw MEG data^71^. The point-spread function of the beamformer used in this study has been clearly shown by Sekihara^72^,^73^. In the present study, the σ_c_ value was 0.36σ_1_ (see Supplementary material for more information).

The target of the virtual channels was the cM1 gyrus. Using Montreal Neurological Institute (MNI) coordinates, 40 virtual channels were selected in cM1 with an inter-sensor spacing of approximately 2.5 mm (Fig. 6B). Then, the virtual channel location coordinates on individual MRIs were extracted using MNI coordinates and warping parameters calculated with the help of the Statistical Parametric Mapping 8 program (SPM8, Wellcome Department of Imaging Neuroscience, London, UK) using an MRI-T1 template and individual MRI-T1 images. This procedure was applied for statistical analysis in accordance with the same MNI coordinates across subjects. A tomographic reconstruction of data was created by generating a single-sphere head model based on the shape of the head obtained from the structural MRI of each participant. The location of the virtual channel in cM1 converted into individual coordinates from MNI were visually confirmed in all subjects. In order to contrast the cM1 signals, the following seven ROIs were also extracted: cS1, c-frontal, c-parietal, iM1, iS1, i-frontal, and i-parietal. In each ROI, 40 virtual channels were extracted with an intersensor spacing of approximately 2.5 mm (Fig. S2).

Stimulus-locked analyses are not appropriate for investigating relationships between real and imagined movements because any difference in the amplitude between the two movements could be due to a latency jitter in motor-related components. Such jitter can be caused by the fact that people respond at different times. To adjust the latency jitter in this study, we used the basic Woody technique^74^,^75^. This technique calculates cross-correlation between a single-trial waveform and the trial-averaged waveform (template) and shifts the latency of each single-trial waveform to that of its maximum correlation with the template. Although this technique was originally developed as an alternative averaging method for ERP analysis and many have used this technique to clarify brain function^76^,^77^,^78^, a recent study has extended its use to single-trial analysis^79^. In the present study, cross-correlations of waveforms between the single-trial waveform and template were calculated for each cM1 virtual channel from −500 to 500 ms (from the presentation of the execution cue). Each single-trial waveform was shifted using the latency with maximum correlation among all virtual channels (Table S1). Because the latency of the execution cue on each shifted single-trial waveform was different, we defined the time corresponding to the execution cue on the averaged waveform in each shifted waveform as a “response onset time.” The response onset time was set as 0 ms, and all time windows were relative to this time. After that, the baseline was set from −3500 to −3000 ms. Data from each epoch were normalized by subtracting the means and then dividing them by the SD of the baseline values. The normalized amplitude of each cM1 virtual channel from −2000 to 1000 ms was then resampled over an average 50-ms time window, sliding by 50 ms (61 time points in total).

### Intensity of M1 activity during real and imagined movements

To compare the intensity of cM1 activity between real and imagined movements, we calculated the root mean square (RMS) from averaged normalized waveforms of all cM1 virtual channels for both movements. The RMS analysis shows the activity of a selected area and effectively evaluates the global neural activity^80^. For both real and imagined movements, RMS amplitudes were averaged over the three movement types. Then, these averaged RMS amplitudes were statistically compared between real and imagined movements using the Mann–Whitney U-test. The intensity of brain activity between real and imagined movements was also compared in the other seven ROIs.

### Decoding analyses

Each of the consecutive four time points of normalized amplitudes (200-ms time window) in all left cM1 virtual channels was used as a decoding feature to classify the movement type from −2000 to 1000 ms with a 50-ms overlap. This time window was selected to obtain a high decoding accuracy and a detailed temporal fluctuation of decoding accuracy as previously reported^81^. A support vector machine operating on the MATLAB 2013a software (MathWorks, Natick, MA, USA), extended to discriminate multiple movements^82^, was used to classify the movement type. Decoding accuracy was evaluated using a 10-fold cross-validation. Each dataset was divided into 10 parts; classifiers were determined from 90% of the dataset (training set) and were tested on the remaining 10%, so that the testing dataset was independent of the training dataset for each time point. This procedure was then repeated 10 times. The averaged decoding accuracy over all runs was used as a measure of decoder performance. The binomial test was used to confirm that the decoding performance significantly exceeded chance levels. In addition to the above analysis, decoding accuracy was calculated in each cM1 virtual channel to examine which contains important information for representing the movement type. This single virtual channel-based decoding analysis was also performed in the other seven ROIs. Moreover, to examine the effect of frequency powers on decoding accuracy, decoding analysis was conducted in cM1 and other seven ROIs using the following frequency powers: alpha (8–13 Hz), beta (13–25 Hz), low gamma (25–50 Hz), and high gamma (50–100 Hz) bands. The power of each frequency band for each virtual channel was calculated using a fast Fourier transform for each 500-ms signal.

### Comparison of decoding accuracy between real and imagined movements

To examine the similarities and differences in the cM1 signals representing motor information between real and imagined movements, spatial and temporal correlations of decoding accuracy between the two movements were calculated. For spatial correlation, spatial distribution of decoding accuracies between real and imagined movements was analyzed using Pearson’s correlation test at each time point. Then, the correlation coefficient was averaged over subjects at each time point. In addition, using Spearman’s rank correlation test, temporal correlation was calculated at each 500-ms time window from −1000 to 500 ms, sliding by 50 ms, in all combinations of virtual channel pairs. The correlation coefficient at each time window was then averaged over subjects. The results of the temporal correlation analysis were corrected for multiple comparisons using FDR. Spatiotemporal correlations were also calculated in the other seven ROIs using the same procedure.

## Additional Information

**How to cite this article**: Sugata, H. *et al.* Common neural correlates of real and imagined movements contributing to the performance of brain-machine interfaces. *Sci. Rep.* **6**, 24663; doi: 10.1038/srep24663 (2016).

## Supplementary Material

Supplementary Movie 1

Supplementary Movie 2

Supplementary Movie 3

Supplementary Movie 4

Supplementary Movie 5

Supplementary Information
