# Supplementary material for: Common neural correlates of real and imagined movements contributing to the performance of brain–machine interfaces
Source: Sci Rep. 2016 Apr 19;6:24663. doi: 10.1038/srep24663 (PMC4835797; doi:10.1038/srep24663)
Supplement: Supplementary Information [file srep24663-s6.pdf]

Supplementary Information for

Common neural correlates of real and imagined movements contributing to the performance of brain–machine interfaces

Hisato Sugata [1,2], Masayuki Hirata [1,3], Takufumi Yanagisawa [1,3,4], Kojiro Matsushita [1,5], Shiro Yorifuji [3] and Toshiki Yoshimine [1]

[1] Department of Neurosurgery, Osaka University Medical School, 2-2 Yamadaoka, Osaka, 565-0871, Japan

[2] Faculty of Welfare and Health Science, Oita University, 700 Dannoharu, Oita, 870-1192, Japan

[3] Division of Functional Diagnostic Science, Osaka University Graduate School of Medicine, 1-7 Yamadaoka, Osaka, 565-0871, Japan

[4] ATR Computational Neuroscience Laboratories, 2-2-2 Hikaridai, Keihannna Science City, Kyoto, 619-0288, Japan

[5] Department of Mechanical Engineering, Gifu University, 1-1 Yanagido, Gifu, 501-1193, Japan

Contents:

Supplementary Figures 1-9
Information for Supplementary Movies 1-5
Supplementary Table 1
Supplementary Materials

Supplementary Figures

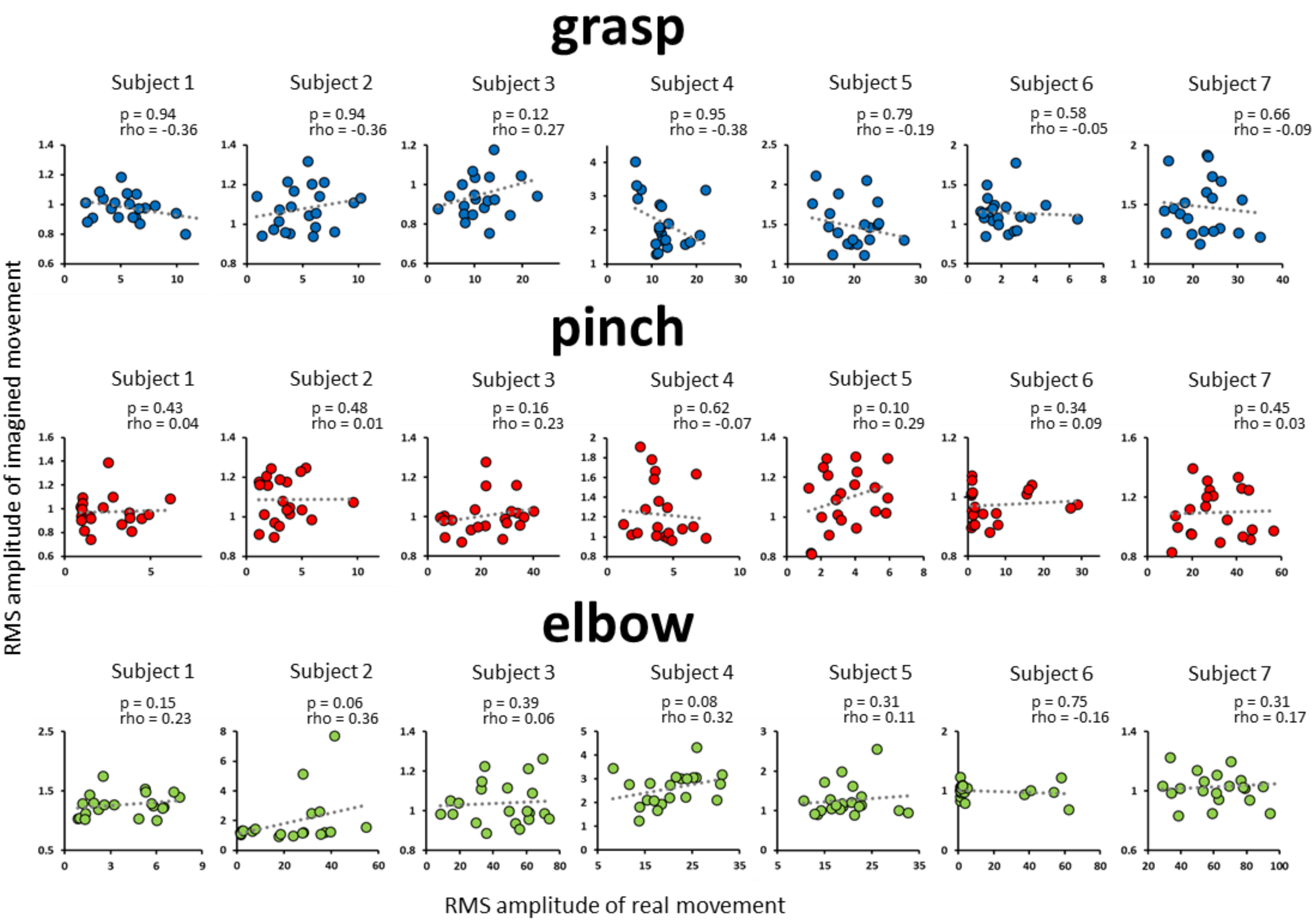

Figure S1. Scatter diagrams showing Electromyogram (EMG) activity for real and imagined movements. EMG analysis was performed on seven subjects who showed clear EMG activity for real movements and no environmental noise contamination in any trial. There was no significant positive correlation between EMG signals recorded during the real and imagined movements in any of the seven subjects.

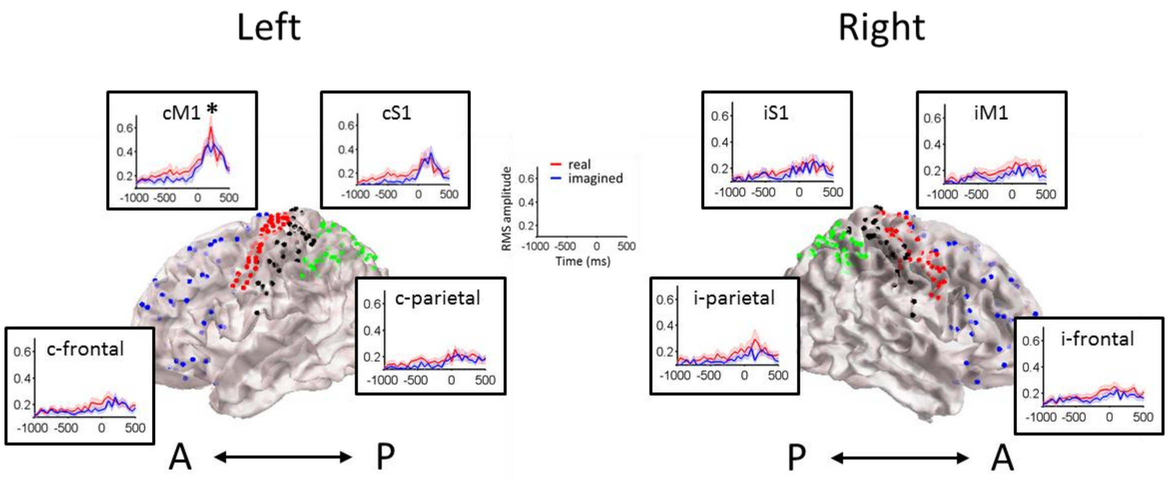

Figure S2. Location of virtual channels and brain activity for real and imagined movements in eight ROIs. The ROIs are indicated by blue dots (frontal areas), red dots (M1), black dots (S1), and green dots (parietal areas). Although the intensities of brain activity increased during both real and imagined movements in all ROIs, there was no significant difference between the two movements in any area except in cM1 (Mann–Whitney U-test, * $p < 0.01$). Red and blue shaded areas indicate standard error for real and imagined movements, respectively. A, anterior; P, posterior; c, contralateral; i, ipsilateral

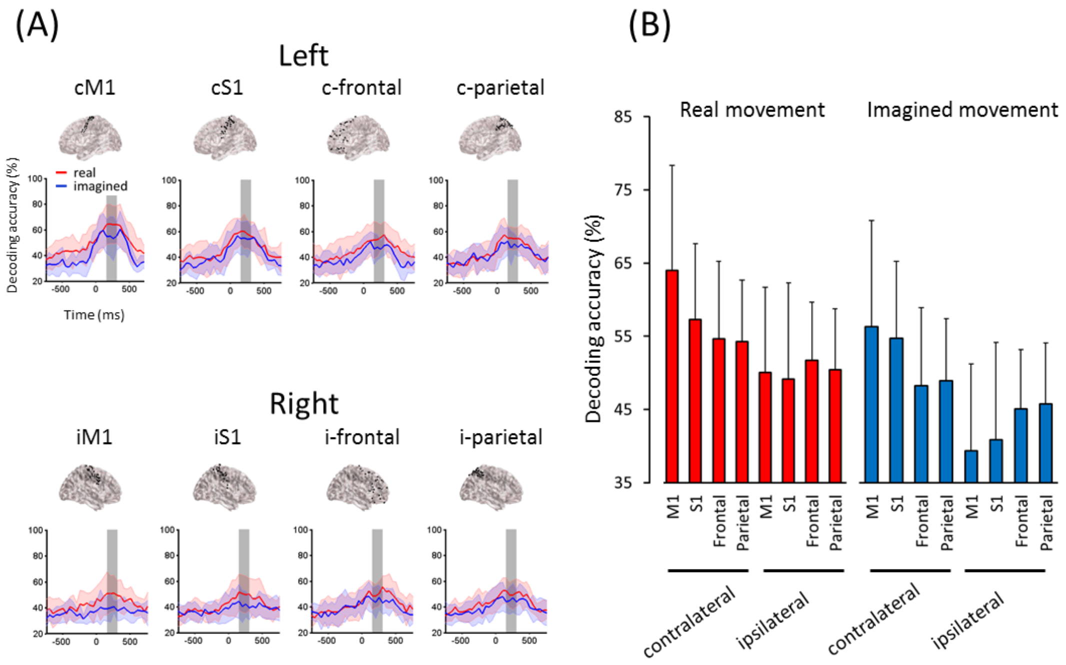

Figure S3. A: Time course of decoding accuracy in eight ROIs. Decoding accuracy increased in all ROIs after response onset in both real and imagined movements. Red and blue shaded areas indicate standard deviation (SD) for real and imagined movements, respectively. **B:** Time-averaged decoding accuracy from 150 to 300 ms in the eight ROIs (vertical gray-shaded areas in **A**). The highest decoding accuracy was obtained in cM1 followed by the second highest in cS1 for both movement types (error bar, SD). c, contralateral; i, ipsilateral

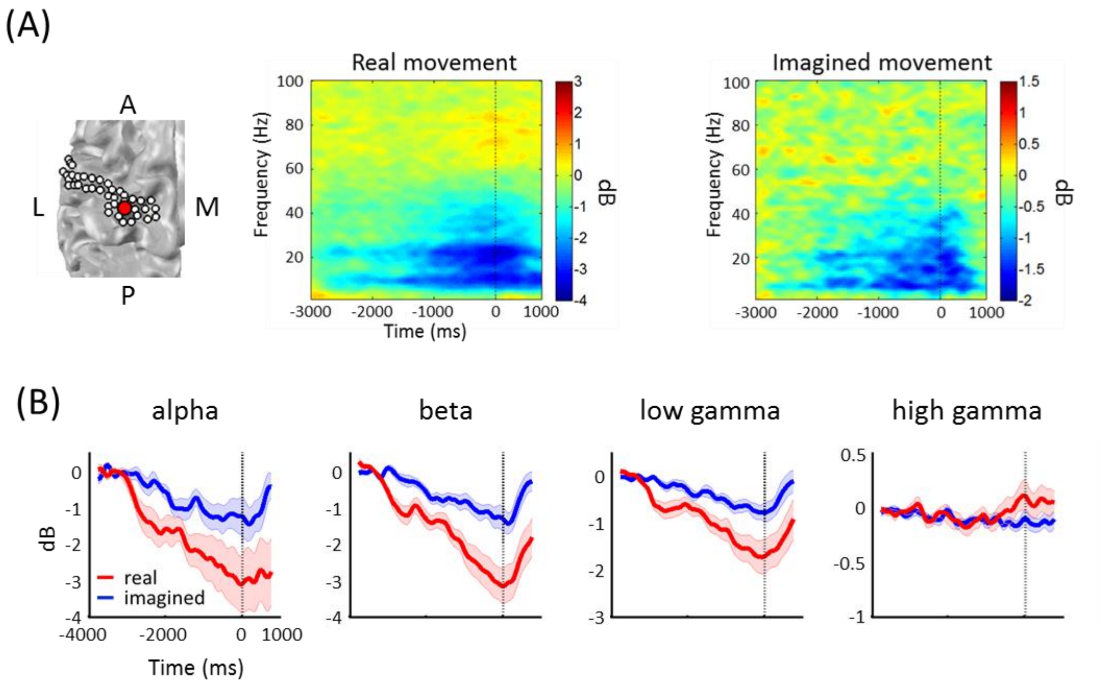

Figure S4. A: Time frequency maps averaged over subjects and movement types during real and imagined movements. The red circle in the upper left panel indicates the M1 virtual channel used in the plots (channel number 11: x = −30, y = −22, and z = 72 in MNI space). Clear event-related desynchronizations (ERDs) were observed from 5 to 50 Hz before response onset for both real and imagined movements. Event-related synchronization (ERS) was observed around 80 Hz in line with the response onset for real movement. **B:** Time course of the power changes in the alpha (8–13 Hz), beta (13–25 Hz), low gamma (25–50 Hz), and high gamma (50–100 Hz) bands calculated from the time frequency maps. ERDs in the alpha, beta, and low gamma bands negatively peaked in line with the response onset for both real and imagined movements. Conversely, ERS in the high gamma bands positively peaked, corresponding to the response onset for real movement.

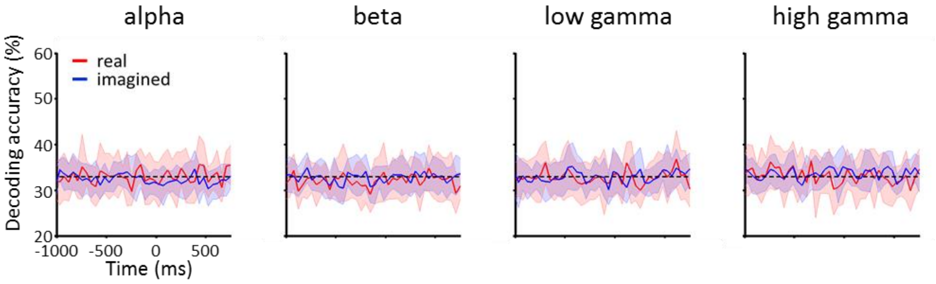

Figure S5. Time course of decoding accuracy in cM1 calculated using alpha (8–13 Hz), beta (13–25 Hz), low gamma (25–50 Hz), and high gamma (50–100 Hz) frequency powers. No significant decoding accuracies were obtained. Red and blue shaded areas indicate SD for real and imagined movements, respectively. Horizontal dotted lines indicate chance level (33.3%).

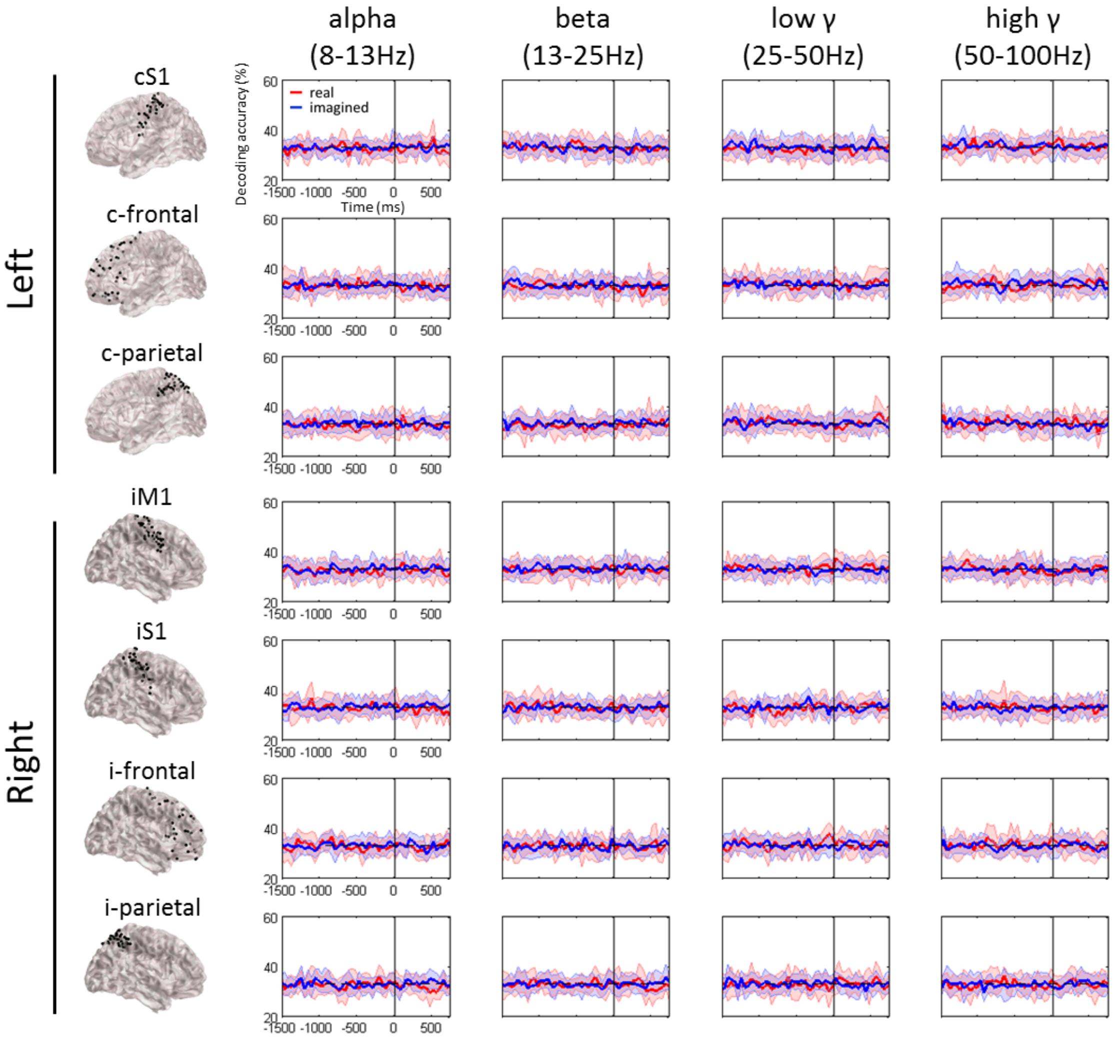

Figure S6. Time course of decoding accuracy in seven ROIs calculated using alpha (8–13 Hz), beta (13–25 Hz), low gamma (25–50 Hz), and high gamma (50–100 Hz) frequency powers. No significant increase in decoding accuracy was observed in any of the seven ROIs. Red and blue shaded areas indicate SD in real and imagined movements, respectively. c, contralateral; i, ipsilateral

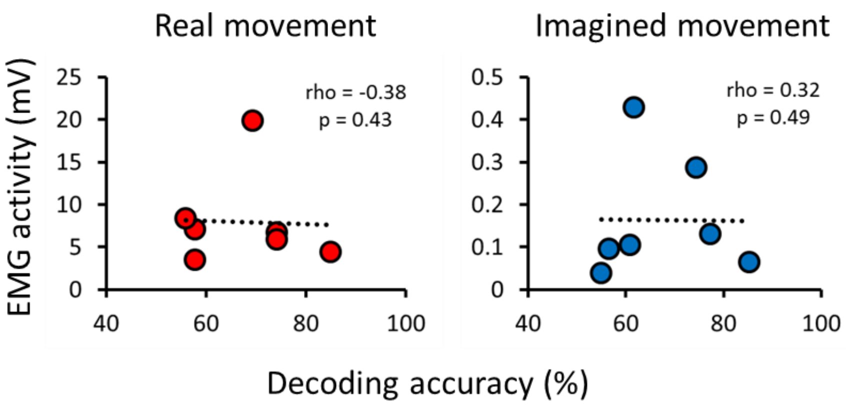

Figure S7. Scatter diagrams showing peak EMG activity and decoding accuracy for real and imagined movements. Correlation analysis was performed for seven subjects who showed clear EMG activity for real movement and no environmental noise contamination in any trial. There was no relationship between EMG activity and decoding accuracy for both movement types (Spearman's rank correlation test, real movement, $p = 0.43$; imagined movement, $p = 0.49$).

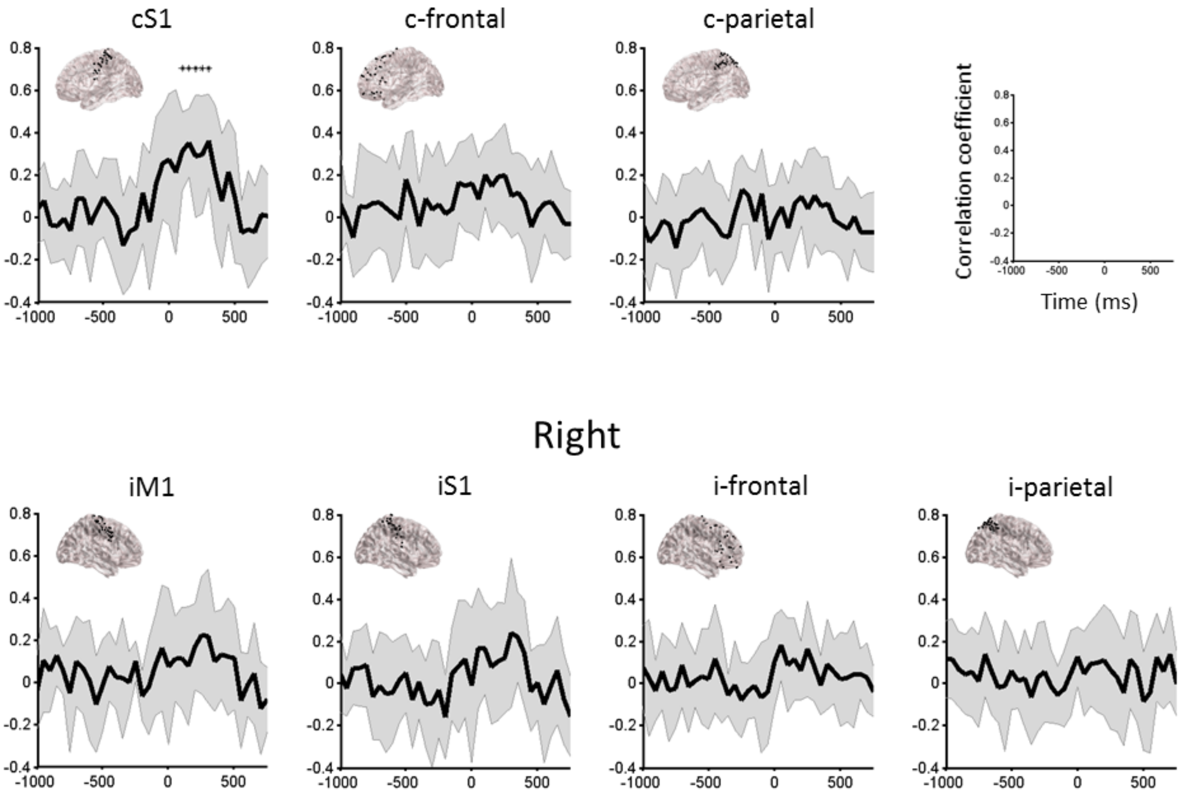

Figure S8. Spatial correlation of decoding accuracy between real and imagined movements averaged over subjects in seven ROIs. Significant spatial correlations were observed only in cS1 after response onset (Pearson's test, * $p < 0.05$). Gray shaded areas indicate SD. c, contralateral; i, ipsilateral

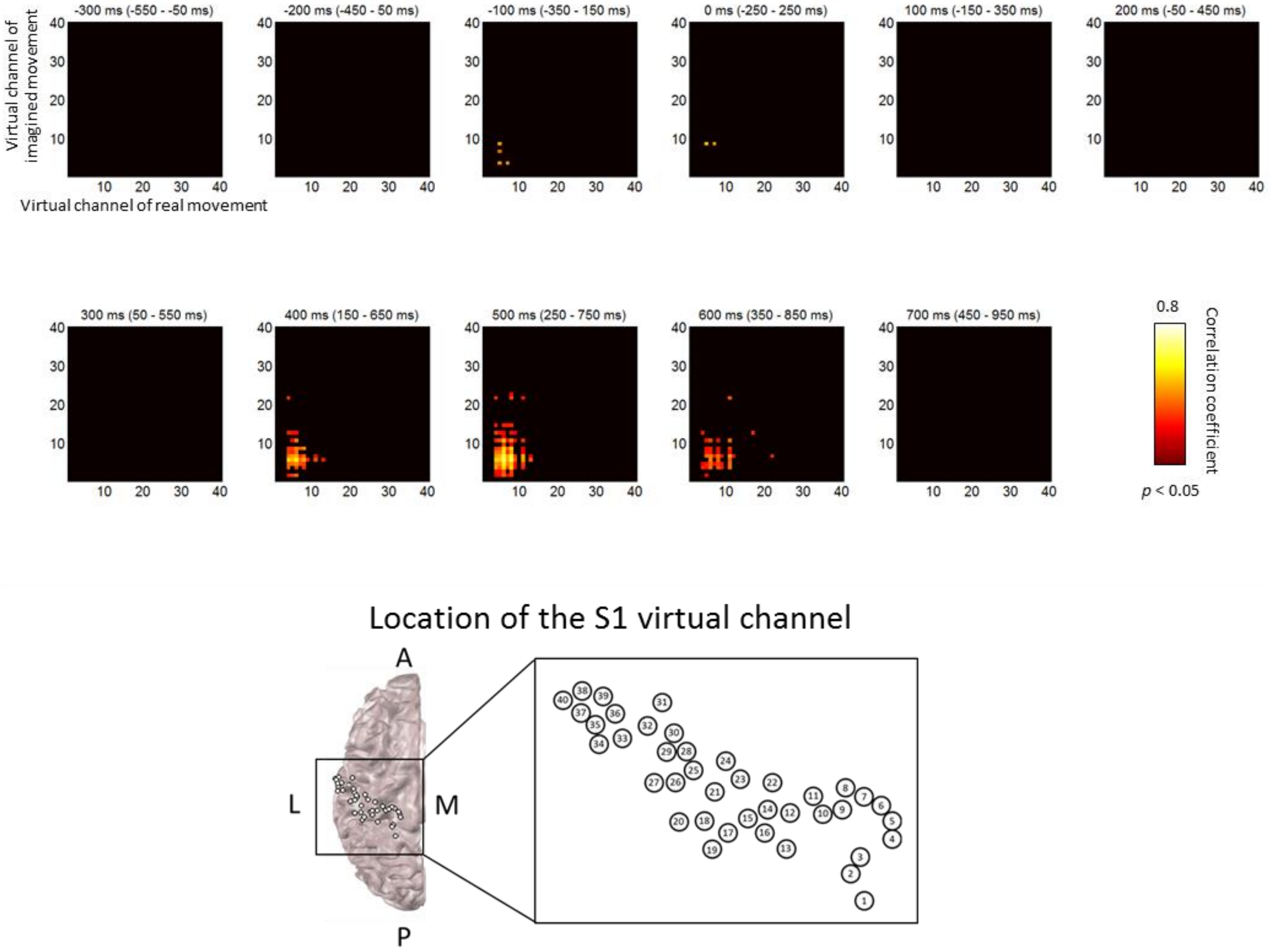

Figure S9. Temporal correlation of decoding accuracy between real and imagined movements in cS1. Each plot depicts the correlation coefficients averaged over subjects at each time window. Temporal correlation began to significantly increase from −100 ms (−350–150 ms) around the medial part of cS1, particularly the hand and arm areas. These significant correlations disappeared near response onset and reappeared from 400 ms (150–650 ms). Correlation coefficients at $p < 0.05$ were considered statistically significant and plotted (Spearman's rank correlation test, $p < 0.05$, FDR-corrected). A, anterior; L, lateral; M, medial; P, posterior

Information for Supplementary Movies

Supplementary movie 1. Time course of decoding accuracy averaged over subjects in cM1 virtual channels during the real movement. Decoding accuracies were significantly increased after response onset around the hand and arm areas; it was plotted for the first sample acquired in the time window. Virtual channels with a significant accuracy are marked with an “x” (binomial test, $p < 0.01$).

Supplementary movie 2. Time course of decoding accuracy averaged over subjects in cM1 virtual channels during the imagined movement. Decoding accuracies were significantly increased after response onset around the hand and arm areas; it was plotted for the first sample acquired in the time window. Virtual channels with a significant accuracy are marked with an “x” (binomial test, $p < 0.01$).

Supplementary movie 3. Time course of decoding accuracy averaged over subjects in all ROIs during real movement. Decoding accuracy increased in cM1 and cS1 after response onset around the hand and arm areas. The first sample acquired in the time window was plotted.

Supplementary movie 4. Time course of decoding accuracy averaged over subjects in all ROIs during imagined movement. Decoding accuracy increased in cM1 and cS1 after response onset around the hand and arm areas. The first sample acquired in the time window was plotted.

Supplementary movie 5. Temporal correlation of decoding accuracy between real and imagined movements in cM1. Correlation coefficients were averaged over subjects. Correlations began to significantly increase from −200 ms (from −450 to 50 ms) around the medial part of cM1, particularly the hand and arm areas. These significant correlations disappeared once around response onset and began to reappear from 400 ms (150–650 ms). Correlation coefficients at $p < 0.05$ were considered statistically significant and were plotted (Spearman’s rank correlation test, FDR-corrected).

Table S1.

Table 1 Mean latency (ms) with maximum correlation in each subject							
subject	real movement				imagined movement		
	elbow	grasp	pinch		elbow	grasp	pinch
S1	214.6	174.7	245.8		149.9	205.8	139.2
S2	198.5	184.6	194.7		216.3	243.7	252.9
S3	197.7	305.4	193.2		238.9	194.6	225.8
S4	234.8	238.7	183.7		210.3	190.7	246.6
S5	253.8	225.9	249.5		245.2	213.6	243.9
S6	128.1	207.7	180.6		186.9	256.6	209.4
S7	154.0	91.1	131.5		203.4	160.9	238.8
S8	186.3	262.2	180.5		200.1	139.9	247.9
S9	208.6	225.1	196.2		136.4	220.1	219.7
S10	219.6	200.7	212.1		249.2	192.9	196.2
mean	199.6	211.6	196.8		203.7	201.9	222.1
SD	35.0	54.0	32.2		36.0	33.2	32.7

SD; Standard Deviation

There was no significant difference among movement types and between real and imagind movements.

Supplementary Materials

In the present study, we calculated σ_c to show the point-spread function of the beamformer analysis from a single subject. First, we calculated the signal-to-noise ratio (SNR) from a signal of M1 virtual channel (channel number 11, x = −30, y = −22, z = 72, in MNI space, gray arrow and red circle in the figure below) using the snr.m function of MATLAB. The time range from −1000 to 0 ms was defined as the background noise signal, and the range from 0 to 1000 ms was defined as the target signal. The results showed that the SNR in the present study was 2.8 dB. Then, we calculated the σ_c using the following formula: SNR = σ_1/σ_c. More information regarding the point-spread function of the beamformer has been provided by Dr. Sekihara [1,2].

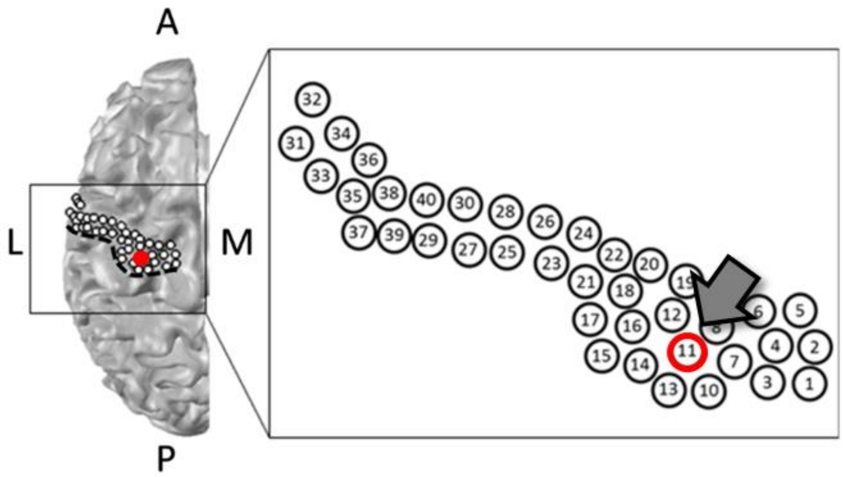

EMG analysis was performed for seven subjects who showed clear EMG activity for real movements and no environmental noise contamination in any trial. First, EMG activities were bandpass filtered at 1.5–100 Hz and averaged over trial and movement type for both real and imagined movements. Then, the data were normalized by subtracting the means and then dividing them by the SD of the baseline values (−3500–−3000 ms from the response onset). Subsequently, RMS amplitudes of normalized EMG activities were calculated for both real and imagined movements and downsampled to 40 Hz. Then, the correlation coefficient of RMS activities between real and imagined movements were calculated using the 20 time points of the RMS amplitudes (−500–500 ms from the response onsets).

References

1 *Sekihara, K. & Nagarajan, S. S. Adaptive Spatial Filters for Electromagnetic Brain Imaging. (Springer-Verlag Berlin Heidelberg, 2008).*

2 *Sekihara, K., Sahani, M. & Nagarajan, S. S. Localization bias and spatial resolution of adaptive and non-adaptive spatial filters for MEG source reconstruction. Neuroimage 25, 1056-1067, doi:10.1016/j.neuroimage.2004.11.051 (2005).*
